# Adaptation of the layer V supraspinal motor corticofugal projections from the primary (M1) and premotor (PM) cortices after CNS motor disorders in non-human primates: A survey

**DOI:** 10.1515/tnsci-2022-0342

**Published:** 2024-06-07

**Authors:** Eric M. Rouiller

**Affiliations:** Department of Neurosciences and Movement sciences, Section of Medicine, Faculty of Sciences and Medicine, University of Fribourg, Ch. du Musée 5, CH-1700 Fribourg, Switzerland

**Keywords:** monkeys, motor functional recovery, tract tracing

## Abstract

Motor commands are transmitted from the motor cortical areas to effectors mostly via the corticospinal (CS) projection. Several subcortical motor nuclei also play an important role in motor control, the subthalamic nucleus, the red nucleus, the reticular nucleus and the superior colliculus. These nuclei are influenced by motor cortical areas via respective corticofugal projections, which undergo complex adaptations after motor trauma (spinal cord/motor cortex injury) or motor disease (Parkinson), both in the absence or presence of putative treatments, as observed in adult macaque monkeys. A dominant effect was a nearly complete suppression of the corticorubral projection density and a strong downregulation of the corticoreticular projection density, with the noticeable exception in the latter case of a considerable increase of projection density following spinal cord injury, even enhanced when an anti-NogoA antibody treatment was administered. The effects were diverse and less prominent on the corticotectal and corticosubthalamic projections. The CS projection may still be the major efferent pathway through which motor adaptations can take place after motor trauma or disease. However, the parallel supraspinal motor corticofugal projections may also participate in connectional adaptations supporting the functional recovery of motor abilities, representing potential targets for future clinical strategies, such as selective electrical neurostimulations.

## 1 Background and aim

The control of voluntary movements in primates depends to a large extent on corticospinal (CS) projections originating from multiple fronto-parietal cortical areas, representing parallel CS motor command modules [1,2,3,4,5]. However, parallel motor corticofugal projections originating from layer V and terminating at various supraspinal levels, for instance in the subthalamic nucleus, the red nucleus, the tectum, and the reticular formation, also play a role in motor control [3,4,6,7,8,9,10,11]. In previous studies conducted in our laboratory, the densities of corticosubthalamic [12], corticorubral [13], corticotectal [14] and corticoreticular [15] projections were quantified in intact adult macaque monkeys. 1All experiments in macaque monkeys were covered by official veterinary authorizations (see for detail the related publications from our laboratory listed in this paragraph). Subsequently, in separate recent reports, the changes in the densities of these corticofugal projections induced by cortical or spinal trauma or in the case of Parkinson symptoms were assessed [12,13,16,17] (see footnote 1). These changes were rather disparate and also varied according to different putative treatments aimed at enhancing functional recovery from the respective motor disorders. The goal of the present review article is to provide here a summarized, comprehensive and synthetic survey of these complex data, previously reported in a fragmented manner across several publications. The observed changes in projections densities in experimental animals may represent an adaptation of the motor system to support motor functional recovery after injury or in case of disease, also possibly enhanced by a specific therapy.

## 2 Methodology

In previously published studies from our laboratory [12,13,14,15,16,17], the anterograde tracer biotinylated dextran amine (BDA) was injected unilaterally either in the premotor cortex (PM) or in the primary motor cortex (M1) of adult macaque monkeys divided into four groups:

**– Intact monkeys** (BDA injections in PM or in M1);
**– SCI**: monkeys subjected to a spinal cord injury consisting of a near hemisection at cervical level C7–C8 (BDA injections in the contralesional M1 only);
**– MCI**: monkeys subjected to an unilateral injury (lesion) of the M1 hand area (BDA injections in the homolateral PM only); and
**– PD**: monkeys subjected to Parkinson-like symptoms induced by MPTP infusion (unilateral BDA injections in PM or in M1).

The multi-site BDA injections in PM were widespread, covering a large part of the caudal PMd and PMv (or areas F2 and F4), also encroaching some part of the rostral PMd and PMv (or areas F7 and F5), except one monkey in the MCI group in which BDA was injected in PMd (areas F2 and F7). The multi-site BDA injections in M1 were targeted mostly to the hand area, with some spread to more proximal forelimb territories (wrist, elbow). The precise position and extent of all BDA injection sites in PM or in M1 were represented graphically in recent reports, as well as individual data for the BDA volumes injected and the number of injection sites, listed in the form of Tables [12,15,16]. In the SCI, MCI, and PD groups, the BDA injections took place between 3 and 6 months postinjury or MPTP infusion, a time window allowing post-lesion behavioral, electrophysiological, and pharmacological investigations, in particular, to assess the time course and possible mechanisms of (incomplete) functional recovery of motor abilities, with emphasis on manual dexterity.

**Figure 1:**
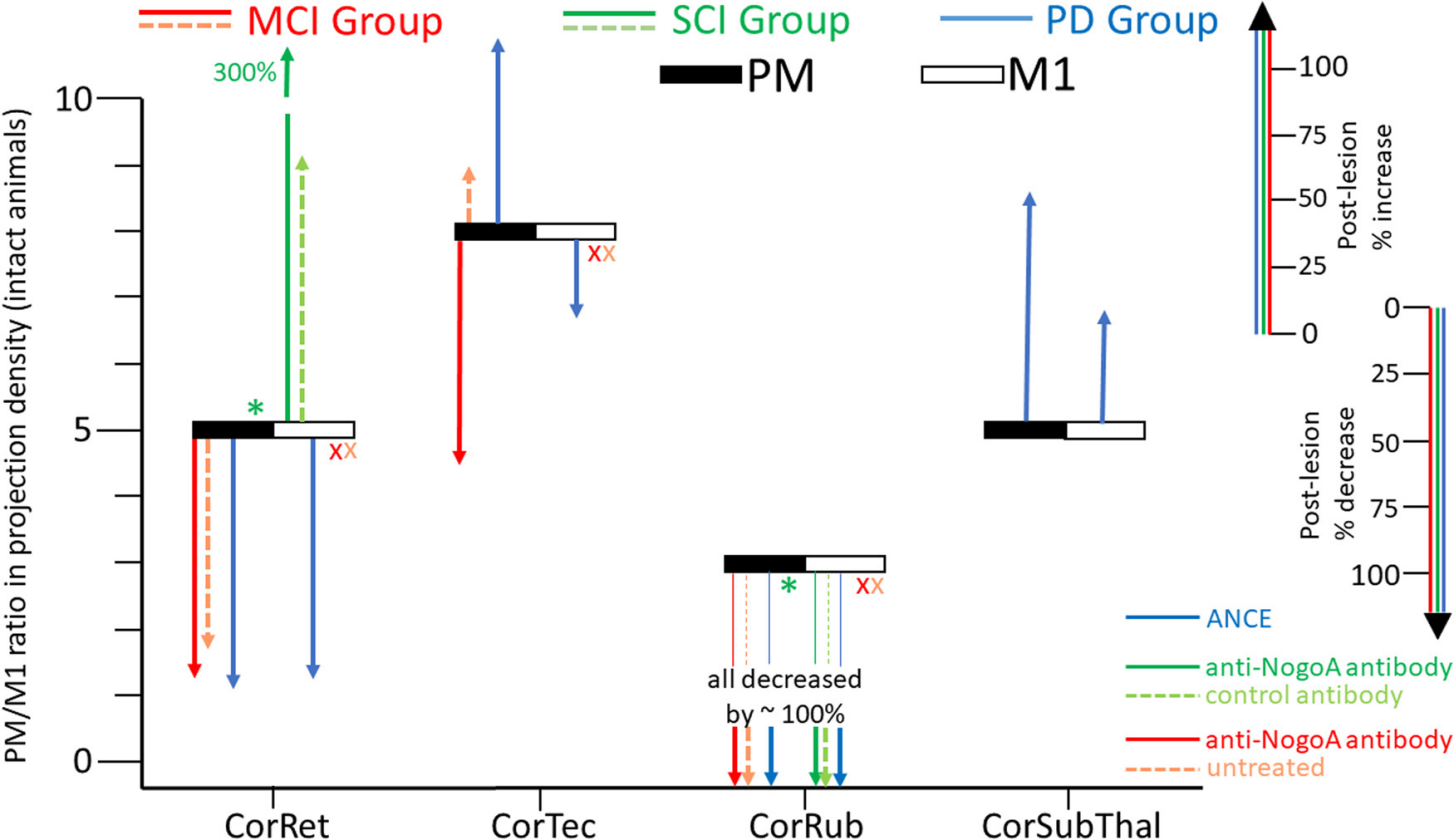
The 4 corticofugal projections (abscissa), namely corticoreticular (CorRect), corticotectal (CorTec), corticorubral (CorRub) and corticosubthalamic (CorSubThal), were quantified in intact monkeys and are represented here by black rectangles when originating from the premotor cortex (PM) and white rectangles when originating from the primary motor cortex (M1). **The position of the black/white rectangles along the ordinate corresponds to the ratio in projections densities in the respective target regions when originating from PM divided by that originating from M1 (PM/M1).** All four projections are denser when originating from PM than from M1 (range 3-8x denser; see text). The experimental groups (MCI, SCI, PD) are represented in red, green and blue, respectively: Spinal Cord Injury (SCI); M1 lesion (MCI); Parkinson symptoms (PD). In each experimental group, a therapy was tested. In MCI, a subgroup of monkeys was treated with the anti-NogoA antibody (solid red arrows), compared with an untreated subgroup of monkeys (dashed light red arrows). In SCI, a subgroup was treated with the anti-NogoA antibody (solid green arrows), compared with a subgroup of monkeys in which a control antibody was administered (dashed light green arrows). Each group/subgroup of monkeys comprised 2–3 animals, which can be identified individually in the Tables published in previously published articles [12,13,14,15,16,17]**. The vertical colored arrows directed upwards indicate an increase of the corresponding projection density as a result of the lesion/disease, in the absence or in the presence of the applied treatment, expressed in % with respect to the corresponding density in intact animals (ordinate at the right). Reciprocally, vertical arrows directed downward indicate a decrease of the corresponding projection density (ordinate at the right).** Colored arrows positioned on the black rectangle are for projections originating from PM, on the white rectangles for those originating from M1. Note that in the PD group, all monkeys were treated with the ANCE cellular therapy (no control subgroup see reference [28]). The red x symbols mean that in the MCI group, only PM was injected with BDA as M1 was lesioned. The green * symbols indicate that in the SCI group, only M1 was injected with BDA (no BDA injection in PM available in the SCI group). Note that the corticosubthalamic projection was investigated in intact monkeys and in PD symptoms monkeys only, as the corticosubthalamic projection is closely related to basal ganglia circuitry, itself affected by PD. Moreover, the corticotectal projection was not analyzed in the SCI group, because the superior colliculus region was not included in the tissue blocks available for histological analysis. References [12,13,14,15,16,17] are for the previous studies from which the data have been reprocessed here in percentages of density ratios (Figure 1) in order to offer in the present review article a comprehensive and easier comparison across corticofugal projections and experimental groups, including intact monkeys.

An anti-NogoA antibody therapy [18] was tested in the SCI [19,20,21,22,23] and MCI [23,24] groups and compared with untreated monkeys subjected to the same lesion. In the PD group, all four monkeys were treated with the autologous neural cell ecosystem (ANCE) autologous neural cellular therapy [25,26,27,28,29] (see also legend of Figure 1). In the MCI and SCI groups, previously published behavioral data have demonstrated significantly enhanced functional recovery in the anti-NogoA antibody-treated subgroups as compared to the corresponding untreated subgroups, especially when the manual dexterity task was the most challenging [19,22,23,24]. In addition, the anti-NogoA antibody treatment promoted a faster functional recovery, decreasing the time to reach the plateau of recovery [19,22,23,24].

As previously reported [12,13,14,15,16,17], the BDA anterogradely labelled axonal boutons (*en passant* and *terminaux*) were quantified in four targets of the corticofugal supraspinal projections originating from the layer V of PM or M1, namely the subthalamic nucleus [12], the red nucleus [13], the superior colliculus [14,17] and the reticular formation [15,16]. The goal was to establish the density of the corticosubthalamic, corticorubral, corticotectal and corticoreticular projections, respectively. The total numbers of boutons observed bilaterally in each target area were then normalized based on the number of CS axons labelled with BDA in the same monkey, to allow comparisons of projection densities across monkeys, across cortical areas and across treatments. The normalization procedure was described and discussed in detail in the above-mentioned publications from our laboratory. The original data for each corticofugal projection were published earlier separately [12,13,14,15,16,17]. Note that the corticosubthalamic projection was investigated in the PD group only, whereas the corticotectal projection was not investigated in the SCI group (see legend of Figure 1 for more detail). In the present review article, each group/subgroup of monkeys comprised 2–3 animals, from which the data were reprocessed to derive an average value for the density of the respective corticofugal projection (Figure 1).

The comparison of corticofugal supraspinal projections originating from PM *versus* M1 in intact animals, as well as in different experimental subgroups, is justified by their common status of motor cortical area in the frontal lobe at the origin of CS projections [1,2,3,5]. In addition, it has been shown that PM plays a vicarious role in the functional recovery of motor control following M1 lesion [23,30]. However, in the frontal lobe, CS neurons in M1 are more numerous than in PM [2]. For this reason, the comparison between PM and M1 is based, as described above, on counts of axon terminals in the subcortical targets normalized according to the number of CS axons labelled with the corresponding BDA injection, in the white matter just above the pyramidal decussation [12,13,14,15,16,17].

## 3 Intact adult monkeys

In intact adult macaque monkeys, the corticoreticular, corticotectal, corticorubral and corticosubthalamic projections were denser when originating from PM than from M1, after normalization of the axonal boutons’ numbers with respect to the respective numbers of labelled CS axons. More precisely, as shown in Figure 1, the corticoreticular projection was 5× denser from PM than from M1, whereas this ratio PM/M1 was 8×, 3× and 5× for the corticotectal, corticorubral and corticosubthalamic projections, respectively.

## 4 Experimental monkey groups/subgroups

As shown in Figure 1, the experimental procedures (lesion, disease, treatments) induced a large variety of effects impacting the terminal density of the four corticofugal projections, as compared to intact monkeys. However, the dominating effect was a decrease (downregulation) of the projection densities, especially for the corticorubral and corticoreticular projections. Indeed, the density of corticorubral projections to the red nucleus from both PM and M1 was massively diminished (CorRub in Figure 1), if not totally suppressed (∼−100%) in some cases [13], irrespective of the monkey subgroups and the absence or presence of treatment.

A bit less homogeneous were the impacts observed on the corticoreticular projection densities (CorRet in Figure 1). In the MCI group, the corticoreticular projection densities from PM were decreased, though a bit more in the presence of the anti-NogoA antibody treatment (−90%) than in control monkeys (−80%). In PD monkeys (all ANCE treated), the corticoreticular projection densities were also decreased (about −90%), irrespective of the cortical area of origin (PM or M1). In sharp contrast, the SCI group showed a considerable increase (upregulation) of the corticoreticular projection densities, also more pronounced in the presence of the anti-NogoA antibody treatment (+300%) than in the absence of treatment (control antibody: +100%).

The corticotectal projection (CorTec in Figure 1) exhibited an opposite effect in the PD group, depending on the motor cortical area of origin, with an increase in density when originating from PM (+68%) and a moderate decrease in density when originating from M1 (−27%). Also opposite were the effects in the MCI group, with a modest increase in projection density from PM in the untreated subgroup (+20%), while there was a substantial decrease in density in the anti-NogoA antibody-treated subgroup (−84%).

Finally, the corticosubthalamic projection (CorSubThal in Figure 1) was investigated only in the PD group, in which all monkeys were treated with the cellular therapy ANCE. As a result of MPTP administration inducing PD symptoms [28], and compared to intact monkeys, the densities of the projections from both PM and M1 were increased in the subthalamic nucleus (+83 and +40%, respectively).

## 5 Discussion

Due to obvious limitations inherent to nonhuman primate studies, including ethical concerns, our previous investigations summarized in Figure 1 are partially incomplete, as there is no data for a PD subgroup of monkeys untreated with ANCE (no dashed blue arrows in Figure 1) and no data either for subgroups of SCI monkeys for which the corticofugal projection from PM has not been established (no green arrows issued from PM, as indicated by the green asterisks). Furthermore, the corticosubthalamic projection was investigated in the PD group only, for monkeys treated with ANCE and compared with intact monkeys (no untreated PD subgroup). Ethical regulations also limit the number of non-human primates included in scientific investigations, restricted in the present case to 2–3 animals within each subgroup. Due to all these limitations in terms of low numbers of animals and some missing subgroups of monkeys, the data presented here remain preliminary in that respect.

Nevertheless, the data shown in Figure 1 demonstrate that the supraspinal motor corticofugal projections from PM or M1 are in most cases significantly modified in case of lesion (MCI or SCI) or disease (PD), either in the form of a downregulation (majority) or an upregulation (minority), depending mainly on the pathology and/or less often on the motor cortical area of origin.

In the PD group, in part due to the ANCE treatment, the progressive functional recovery from PD symptoms in the months following the MPTP intoxication [28] appears to be correlated with a density increase of the hyperdirect pathway [8] terminating in the subthalamic nucleus, originating from both PM or M1. This change may represent an adaptation to the pathology and/or to the ANCE treatment in the form of an impact on the basal ganglia circuitry to partly compensate functionally for the loss of dopaminergic neurons in the substantia nigra [28]. In parallel, still in the PD group, the corticorubral and corticoreticular projections from PM and M1 are both strongly decreased, also possibly contributing to the progressive functional recovery from PD symptoms, maybe more indirectly than the adaptation of the corticosubthalamic projections.

In the case of unilateral lesion of M1 (hand area; the MCI group represented by red arrows in Figure 1), there was a general decrease in density for the corticorubral, corticoreticular and corticotectal projections originating from the homolesional PM, possibly representing an anatomical substrate among others for functional recovery, either spontaneous (limited [30]) or enhanced with the anti-NogoA antibody treatment [24]. Pharmacological inactivation experiments have shown that the functional recovery of manual dexterity following unilateral lesion of the M1 hand area depended, at least in part, on the homolateral intact PM, with some more contribution of PMd than of PMv [23,30]. This role of PM in functional recovery thus includes a decrease in PM influence on the superior colliculus, red nucleus, and reticular motor nuclei (downwards red arrows in Figure 1). One may speculate that, on the counterpart, the terminations in the cervical cord of the CS projection from PM are upregulated, by a mechanism of filling the empty space left at the cervical level following the destruction of the M1 CS neurons due to the hand area lesion. In other words, such connectional adaptations may favor the direct influence of PM onto cervical motor circuits, to the expense of an indirect influence of PM via the mesencephalon and brainstem, which is reduced.

Note that after extensive lesion of both M1 and PM, the corticorubral projection from the supplementary motor area (SMA) was reported to be upregulated [31]. The discrepancy with the present results (dowregulation of the corticorubral projection originating from PM) may be explained by the different origin of the corticorubral projection (SMA versus PM) and the extent of the lesion, limited to the hand area in M1 in our case, whereas in [31] the cortical lesion was much bigger in M1 and also included PM.

Finally, in the case of cervical cord hemisection (SCI group represented by green arrows in Figure 1), the density of the corticorubral projections from M1 was massively diminished both in the absence or presence of anti-NogoA antibody treatment whereas, in sharp contrast, the density of the corticoreticular projection was strongly increased. This upregulation of the corticoreticular projection parallels the sprouting of CS axons enhanced by the anti-NogoA antibody treatment [19,20]. Interestingly, the upregulation of the corticoreticular projection was tripled in the presence of the anti-NogoA antibody treatment, possibly representing among other substrates a contribution to the functional recovery, limited in control monkeys and enhanced in treated monkeys [19,22]. The cervical lesion at the C7–C8 level did not induce a degeneration of the CS neurons of origin, but instead a shrinkage of their soma [21], both in the anti-NogoA antibody-treated and control antibody-treated subgroups. The precise position of the C7–C8 lesion, illustrated earlier [16], indicates that the CS tract was nearly completely interrupted, the rubrospinal tract was also strongly affected, whereas the reticulospinal tract was less impacted. Considering that part of the corticorubral and of the corticoreticular projections (also the corticosubthalamic projection) may be collaterals of the CS axons [32,33], the interruption of the CS axons in the spinal cord may trigger a retrograde signal modifying the phenotype of the CS neurons of origin: shrinkage of the soma [21] and the here reported downregulation of the corticorubral projection and upregulation of the corticoreticular projection (green arrows in Figure 1). The opposite effect of the SCI on these 2 projections may be related to the above-mentioned different impacts on the rubrospinal and reticulospinal projections. Of particular interest is the preservation by the SCI of the reticulospinal projection, leaving open the possibility for the motor reticular nuclei to play an important role in the functional recovery of motor control in general, manual dexterity in particular [34,35,36,37,38]. The strong upregulation of the corticoreticular projection after SCI reported here, in the form of a sprouting of axon terminals in motor reticular nuclei, may contribute to reinforce the motor commands originating from M1 in the reticular formation while these commands do not reach the spinal motoneurons anymore because of the lesion interrupting the CS tract. This connectional upregulation is consistent with a previously reported functional recovery after pyramidal lesion involving a marked upregulation in the control of hand movements by the reticulospinal system [34,36]. The downregulation of the corticorubral projection may be interpreted in the sense of a much less important role played in the functional recovery by the red nucleus, due to the significant impact of the SCI on the rubrospinal projection, nearly comparable to the massive impact on the CS tract. In other words, it would make sense to favor the motor reticular nuclei over the red nucleus, by amplifying the transmission of motor cortical commands to the reticular formation and reducing it to the red nucleus.

In the intact animals, it was observed that the density of corticofugal projections (axon terminals) to the reticular motor nuclei, tectum, red nucleus and subthalamic nucleus were 3–8 times stronger when originating from PM than from M1, after normalization (Figure 1). As a significant part of these supraspinal corticofugal projections are axon collaterals from CS axons terminating in the spinal cord [32], it is plausible that the degree of collateralization is higher for CS axons originating from PM than those from M1. If so, this would imply that the corticofugal projections originating from PM in macaques are more reminiscent of the high degree of supraspinal collateralization observed in rodents than those originating from M1, itself at the origin of less supraspinally collateralized CS axons in macaques than in rodents [32].

## 6 Conclusion and future directions

Although the CS projection may be the major efferent pathway through which motor adaptations can take place after motor trauma or disease, the present data support the notion that supraspinal motor corticofugal projections may also play a role in adaptations supporting functional recovery of motor abilities. However, MCI or SCI did not induce similar changes in the corticorubral and corticoreticular projections, as the two types of injury induced a downregulation of the former projection whereas the latter projection was downregulated after MCI but upregulated after SCI. Interestingly, in both cases, the changes were amplified by the administration of the anti-NogoA antibody treatment (solid red or green arrows) as compared to the control subgroups (dashed red or green arrows). Anyway, the major effect of a downregulation of the corticofugal supraspinal projections after lesion can be interpreted as a decrease in the cortical influence on the corresponding motor subcortical nuclei, making the rubrospinal, tectospinal, and reticulospinal projections more independent from motor cortical areas. The clear exception is the corticoreticular projection after SCI which was massively upregulated, thus corresponding to an increased influence of M1 on the reticular formation post-lesion, an effect dramatically enhanced by the anti-NogoA antibody treatment; as a consequence, the reticulospinal projection is more strongly influenced by M1 after SCI. This putative prominent role played by the cortico-reticulo-spinal system in the functional recovery of manual dexterity after SCI, strongly enhanced by the anti-NogoA antibody treatment (upwards green arrows in Figure 1), suggests that mechanisms mobilized for recovery may strongly depend on available residual projections spared by the lesion (or the disease), in the present case the reticulospinal projection, much less impacted by the SCI than the rubrospinal or the direct CS projections. The elaboration of a therapeutic strategy has to take into account a precise assessment not only of impaired brain regions and interrupted fiber tracts but also identify preserved brain areas and spared connections onto which selective treatments may be applied to tentatively reach the best adaptative synergy across the multiple corticofugal projection systems.

Future studies aimed at elucidating the mechanisms of motor functional recovery after trauma or disease need to take into account the complex interactions and adaptations between the multiple parallel motor descending pathways originating from the various motor cortical areas [2,3,5], including the degree of collateralization of the CS axons at various levels of the CNS [32]. Beyond the motor cortical areas and their CS projections, special attention needs to be paid to subcortical motor nuclei which play a crucial role in motor control [7,10,11,34,35,36,37,38,39], especially via the rubrospinal and reticulospinal projections, both in position to be mobilized for functional recovery from motor trauma or disease. Regional selective therapeutical approaches targeting a specific brain region and/or an individual fiber tract are currently under development, both in non-human primates and in human subjects using electrical neurostimulation [40, 41, 42]. In the future, electrical neurostimulation may further be combined with axonal re-growth enhancement strategies and/or cellular therapies.
